# ATM-Dependent Downregulation of USP7/HAUSP by PPM1G Activates p53 Response to DNA Damage

**DOI:** 10.1016/j.molcel.2012.01.021

**Published:** 2012-03-30

**Authors:** Svetlana V. Khoronenkova, Irina I. Dianova, Nicola Ternette, Benedikt M. Kessler, Jason L. Parsons, Grigory L. Dianov

**Affiliations:** 1 Department of Oncology, Gray Institute for Radiation Oncology and Biology, University of Oxford, Roosevelt Drive, Oxford OX3 7DQ, UK; 2 Nuffield Department of Clinical Medicine, University of Oxford, Roosevelt Drive, Oxford OX3 7BN, UK

## Abstract

The deubiquitylation enzyme USP7/HAUSP plays a major role in regulating genome stability and cancer prevention by controlling the key proteins involved in the DNA damage response. Despite this important role in controlling other proteins, USP7 itself has not been recognized as a target for regulation. Here, we report that USP7 regulation plays a central role in DNA damage signal transmission. We find that stabilization of Mdm2, and correspondingly p53 downregulation in unstressed cells, is accomplished by a specific isoform of USP7 (USP7S), which is phosphorylated at serine 18 by the protein kinase CK2. Phosphorylation stabilizes USP7S and thus contributes to Mdm2 stabilization and downregulation of p53. After ionizing radiation, dephosphorylation of USP7S by the ATM-dependent protein phosphatase PPM1G leads to USP7S downregulation, followed by Mdm2 downregulation and accumulation of p53. Our findings provide a quantitative transmission mechanism of the DNA damage signal to coordinate a p53-dependent DNA damage response.

## Highlights

► p53 cellular level is controlled by a specific isoform of USP7 (USP7S) ► Phosphorylation of USP7S at serine eighteen by CK2 is required for USP7S stability ► In response to DNA damage USP7S undergoes ATM-dependent dephosphorylation by PPM1G ► Dephosphorylation of USP7S promotes its degradation and p53 accumulation

## Introduction

The tumor suppressor p53 plays a major role in regulating the cellular response to DNA damage. Although DNA damage occurs in many different ways, the major unprovoked threat is the chemical instability of the DNA molecule caused by the cellular environment, which can result in DNA strand breaks and base modifications. These spontaneous, or endogenous DNA damages, are initiated by intracellular mutagens, such as various reactive oxidative species and other chemically active cellular metabolites (Lindahl, 1993). Consequently, DNA repair is a nonstop process required for maintaining error free DNA replication and transcription. p53 plays a key role in the DNA damage response by coordinating repair processes with DNA replication and also making the decision to undergo programmed cell death (apoptosis) in the case of excessive DNA damage (Levine and Oren, 2009; Vogelstein et al., 2000; Vousden and Lane, 2007). It was recently reported that in cycling cells p53 levels are not stable but are continually changing, through attenuated pulses, in response to the variations in cellular load of DNA damage (Loewer et al., 2010). Mechanistically this is mainly accomplished by up and downregulation of p53 levels by controlling its proteasomal degradation mediated by Mdm2 ubiquitylation (de Rozieres et al., 2000; Kruse and Gu, 2009; Ringshausen et al., 2006; Vousden and Lane, 2007; Vousden and Prives, 2009). USP7 protein (also known as HAUSP) plays a central role in Mdm2 regulation since self-ubiquitylation of Mdm2 promotes its own degradation (Fang et al., 2000; Honda and Yasuda, 2000) that is rescued by formation of a complex between Mdm2 and USP7 proteins (Meulmeester et al., 2005; Tang et al., 2006). In this complex, USP7 promotes continued Mdm2 deubiquitylation and stabilization thus supporting Mdm2-dependent p53 degradation. It has also been shown that disruption of USP7 results in p53 stabilization and inhibition of cell proliferation (Cummins and Vogelstein, 2004; Meulmeester et al., 2005). It is therefore clear that USP7 plays a major role in the DNA damage response by controlling cellular levels of Mdm2 and p53; however, the key question of how USP7 is regulated in response to DNA damage remains unanswered.

Here, we report that in response to DNA damage USP7 is downregulated by the ATM-dependent protein phosphatase PPM1G, thus downregulating Mdm2 and activating the p53 response. Our data reveal an important role for USP7 in DNA damage signal transduction and provide a mechanism coordinating p53 activation.

## Results

### USP7 Is Phosphorylated at Serine 18 by CK2

**Figure 1:**
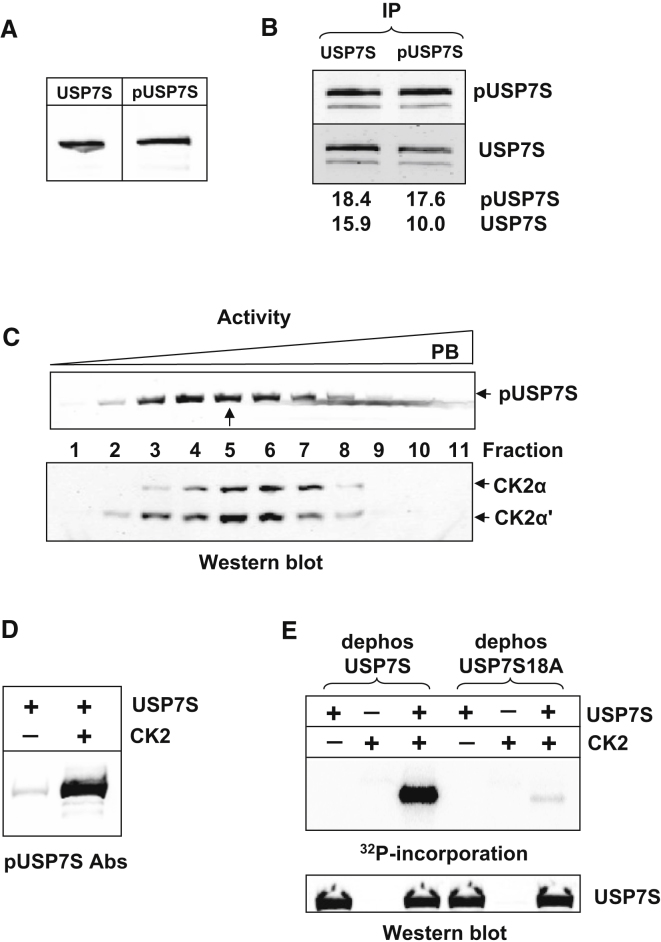
Purification and Identification of CK2 as the Major Protein Kinase Phosphorylating USP7S at Serine 18 (A) HeLa whole-cell extract (40 μg) was separated by 10% SDS-PAGE and analyzed by western blotting using antibodies specific to USP7S or phosphorylated USP7S (pUSP7S). (B) HeLa whole-cell extracts were immunoprecipitated using USP7S or pUSP7S antibodies and analyzed by western blotting. The quantification of bands is shown. (C) In vitro phosphorylation of dephosphorylated recombinant USP7S (1 pmol) by the final hydroxyapatite chromatography fractions purified from HeLa cells, analyzed using pUSP7S (top panel). Western blot analysis of the same fractions with CK2α and CK2α′ antibodies (lower panel). (D) In vitro phosphorylation of dephosphorylated recombinant USP7S (2.8 pmol) by recombinant CK2 protein (2 pmol) was performed at 30°C for 30 min and analyzed using pUSP7S antibodies. (E) In vitro phosphorylation of dephosphorylated recombinant USP7S (1.5 pmol) and dephosphorylated USP7S18A (1.5 pmol) by recombinant CK2 (2 pmol) was performed at 30°C for 60 min in the presence of γ^32^P-ATP and analyzed by 10% SDS-PAGE and phosphorimaging. Equal loading of the substrate is demonstrated using USP7S antibodies (lower panel). See also Figure S1 and Table S1.

It was recently reported that USP7 is phosphorylated at serine eighteen (further referred to as USP7S). However, the biological role of this phosphorylation, and the protein kinase and phosphatase involved in regulation of the serine 18 (Ser18) phosphorylation status of USP7S were not identified (Fernández-Montalván et al., 2007). We hypothesized that phosphorylation of USP7S in response to DNA damage regulates its stability and thus coordinates Mdm2 and p53 activated cellular responses. To uncover the role of Ser18 in USP7S regulation and the proteins involved in Ser18 phosphorylation/dephosphorylation, we generated an antibody specific to phosphorylated and unphosphorylated USP7S (Figures S1A and S1D available online). Both antibodies cross-reacted with USP7S protein in HeLa whole-cell extracts, confirming that phosphorylation of USP7S at Ser18 occurs in human cells (Figure 1A). We further estimated the amount of phosphorylated USP7S existing in human cells by immunoprecipitation of cell extracts using both sets of antibodies. An equal amount of phosphorylated USP7S protein was observed following immunoprecipitation with either USP7S or pUSP7S antibodies specific to phosphorylated protein (Figure 1B and Figure S1E, upper panel). However, pUSP7S antibodies were able to precipitate only 60%–80% of the total USP7S protein (Figure 1B and Figure S1E, lower panel), suggesting that approximately 60%–80% of USP7S is phosphorylated in human cells. We next wished to purify the cellular protein kinase involved in Ser18 phosphorylation. To achieve this, we established an in vitro kinase assay employing recombinant (dephosphorylated) USP7S (Figures S1B and S1C) and used this assay to detect, by western blotting, Ser18 phosphorylation activity in individual fractions after cellular extract fractionation. We observed robust phosphorylation of USP7S at Ser18 that was readily detectable following fractionation of HeLa whole-cell extracts over a series of chromatographic columns (Figures S1F–S1I). Fractions from the final hydroxyapatite column displaying substantial kinase activity against USP7S (Figure 1C, upper panel) were analyzed by mass spectrometry. The major protein kinase found in these fractions was CK2, of which all subunits (CK2α, CK2α′, and CK2β) were detected (Table S1 and data not shown). Western blot analysis of these fractions further confirmed co-purification of CK2 with USP7S Ser18 phosphorylation activity (Figure 1C, lower panel). In fact, USP7S possesses a high score consensus site for CK2 that includes Ser18 (Figure S1A). In accordance with this, we found that recombinant CK2 protein efficiently phosphorylated USP7S (Figure 1D) and that this phosphorylation was specific to Ser18, since when γ-^32^P-ATP was used in an in vitro phosphorylation assay we only observed efficient phosphorylation of previously dephosphorylated wild-type USP7S and not of dephosphorylated USP7S18A mutated protein where Ser18 was substituted for alanine (Figure 1E).

### Phosphorylation of Serine 18 Regulates USP7S Stability and Controls Mdm2 and p53 Levels

**Figure 2:**
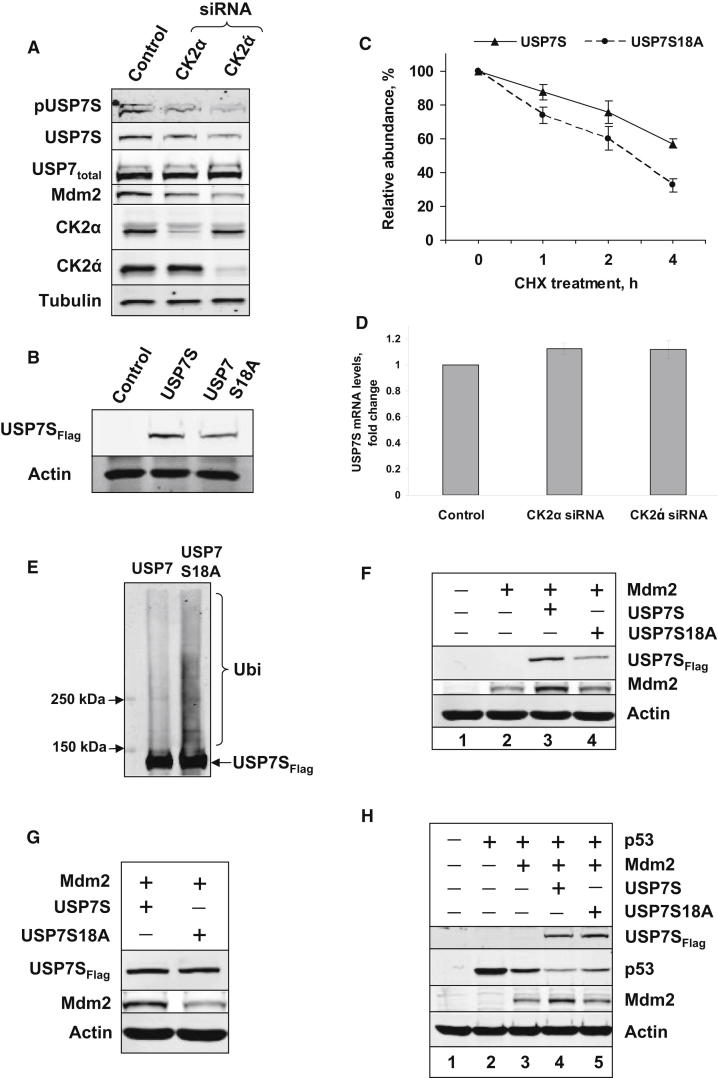
Phosphorylation of Serine 18 Regulates USP7S Stability and Activity (A) HeLa cells were treated with Lipofectamine RNAiMAX transfection reagent in the absence (Control) and presence of CK2α- or CK2α′-specific siRNA (200 pmol) for 72 hr. Cells were pelleted by centrifugation, and whole-cell extracts were prepared and analyzed by western blotting with indicated antibodies. (B) HeLa cells were treated with Lipofectamine transfection reagent in the absence (Control) and the presence of USP7S or USP7S18A expressing plasmid (0.3 pmol) for 24 hr. Cells were pelleted by centrifugation, and whole-cell extracts were prepared and analyzed and western blotting. (C) HeLa cells were treated with Lipofectamine transfection reagent in the presence of USP7S (0.2 pmol) or USP7S18A (0.6 pmol) expressing plasmid for 24 hr. Cells were then treated with 50 μg/ml cycloheximide for 0, 1, 2, or 4 hr and pelleted by centrifugation. Whole-cell extracts were prepared, and the amounts of USP7 were analyzed by western blotting. Statistical data are presented as a mean ± SD of three independent biological experiments. (D) HeLa cells were treated in the presence of CK2α- or CK2α′-specific siRNA as described above, total RNA was prepared and the levels of USP7S and actin mRNA were analyzed by quantitative rtPCR. Statistical data are presented as a mean ± SD of three independent biological experiments. (E) HeLa cells were cotransfected with 1.0 pmol of ubiquitin and 0.5 pmol of either USP7S or USPS18A expressing vectors. After 24 hr, cells were treated with 10 μM MG-132 for 6 hr and pelleted by centrifugation. Equal amounts of whole-cell extracts (500 μg) were then used to pull down Flag-tagged USP7S or USP7S18A using Flag-agarose beads. Immunoprecipitates were using ubiquitin or Flag antibodies. (F) HeLa cells were treated with Lipofectamine transfection reagent in the absence (lane 1) or presence of 0.7 pmol of Mdm2 and 0.2 pmol of either USP7S or USPS18A expression vectors (lanes 3 and 4, respectively). After 24 hr, cells were pelleted by centrifugation and whole-cell extracts were prepared and analyzed by western blotting. (G) Alternatively, HeLa cells were transfected with a 3-fold excess of USP7S18A (0.6 pmol) relative to USP7S expression plasmid. (H) U2OS cells were treated with Lipofectamine transfection reagent in the absence (lane 1) or presence of 0.45 pmol p53, 0.55 pmol Mdm2, and 0.1 pmol USP7S, USPS18A, or USP7ΔN expression plasmids. After 24 hr cells were pelleted by centrifugation, and whole-cell extracts were prepared and analyzed by Western blotting.

To address the cellular role of USP7S phosphorylation by CK2, we transiently knocked down CK2 by small interfering RNA (siRNA) and monitored the phosphorylation status of USP7S. We found that a knockdown of either the CK2α or CK2α′ subunits resulted in reduced USP7S phosphorylation, and decreased amounts of USP7S and Mdm2 proteins (Figure 2A). To verify that USP7S destabilization was due to its reduced phosphorylation and not caused indirectly by inhibition of other CK2 activities, we transiently transfected HeLa cells with equal amounts of plasmid DNA expressing either USP7S or USP7S18A protein, in which Ser18 was substituted by alanine. We found that the USP7S18A protein is indeed less stable compared to the wild-type protein since it had a lower steady state level after expression (Figure 2B) and degraded faster than the wild-type protein after cycloheximide treatment (Figure 2C). To prove that the decline of unphosphorylated USP7S was mainly due to protein degradation rather than downregulation of DNA transcription, we purified total RNA from CK2 knockdown HeLa cells and detected no changes in messenger RNA (mRNA) levels of USP7S in comparison to control cells (Figure 2D). Proteasomal degradation of unphosphorylated USP7S was further confirmed by the higher level of ubiquitylation of the USP7S18A mutant compared to the wild-type protein (Figure 2E). We thus concluded that phosphorylation of Ser18 by CK2 was required for the stability of USP7S. Consequently, as a result of the cellular instability of the USP7S18A protein, it is unable to efficiently maintain the stability of Mdm2 when cotransfected into U2OS cells (Figure 2F). Furthermore, even when cells were transfected with more plasmid expressing USP7S18A to achieve a similar level of protein expression to that of wild-type USP7S, the mutant protein was still unable to efficiently stabilize Mdm2 (Figure 2G), suggesting that Ser18 phosphorylation also affects USP7S deubiquitylation activity. To further demonstrate that modulation of Mdm2 levels by phosphorylated USP7S employed in our study is involved in regulation of cellular p53 levels, we expressed vectors encoding p53, Mdm2 and USP7S in U2OS cells. p53 alone is expressed at high levels (Figure 2H, lane 2), although this was reduced after coexpression with Mdm2 (Figure 2H, lane 3). However, only after coexpression of p53 with Mdm2 and USP7S was the protein level of p53 reduced significantly further (Figure 2H, lane 4), confirming that phosphorylated USP7S efficiently controls the cellular level of p53. In contrast, coexpression with the mutant USP7S18A protein demonstrated the inability of the mutant to efficiently stabilize Mdm2, and consequently was unable to fully suppress p53 expression, in comparison to the wild-type protein (Figure 2H, lane 5).

### USP7 Isoform Phosphorylated at Serine 18 Is the Major Regulator of Mdm2 and p53

**Figure 3:**
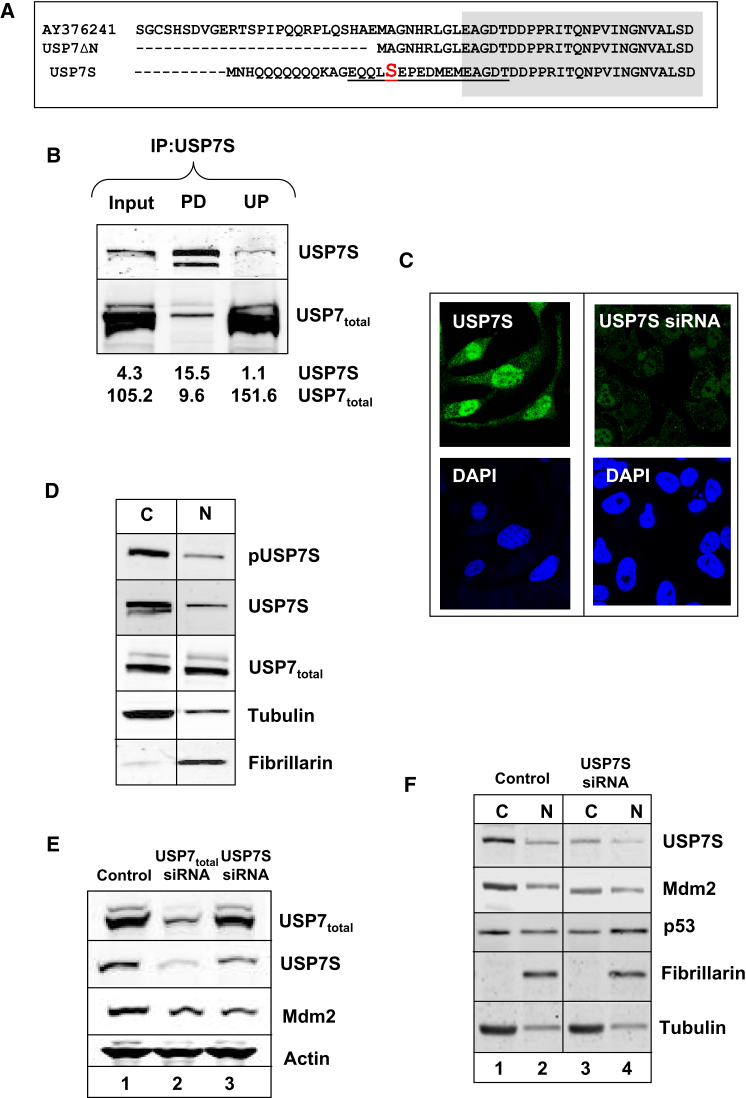
The Serine 18-Containing Isoform of USP7 Is Required for Mdm2 Stability and Concomitant p53 Downregulation (A) N-terminal sequences of the isoforms of USP7, demonstrating the only isoform containing serine 18 (USP7S). Identical sequences are shaded. (B) HeLa whole-cell extracts (Input, 30%) were immunoprecipitated using USP7S antibodies, and the pull-down (PD, 100%) or the unbound proteins (UP, 50%) were separated by western blotting with USP7 antibodies specific to Ser18-containing isoform and antibodies cross-reacting with all the isoforms of USP7 (USP7_total_). (C) HeLa cells were treated with Lipofectamine RNAiMAX transfection reagent in the absence (Control) and presence of USP7S specific siRNA (200 pmol) for a further 48 hr and then immunostained with pUSP7S antibodies followed by Alexa Fluor 488 anti-rabbit IgG. (D) Cytoplasmic (C) and nuclear (N) fractions were prepared from HeLa cells and 40 μg of protein in the C fraction and an equal volume of the N fraction were separated by 10% SDS-PAGE and analyzed by western blotting with USP7S, pUSP7S, and USP7_total_ antibodies. Tubulin and fibrillarin were used as cytoplasmic and nuclear markers, correspondingly. (E) HeLa cells were treated with Lipofectamine RNAiMAX transfection reagent in the absence (Control) and presence of USP7_total_ or Ser18-containing USP7-specific siRNA (200 pmol) for 72 hr. Cells were pelleted by centrifugation, and whole-cell extracts were prepared and analyzed western blotting. (F) HCT116 p53^+/+^ cells were treated with Lipofectamine RNAiMAX transfection reagent in the absence (Control) or presence of USP7S siRNA (200 pmol) for 72 hr. Cells were pelleted by centrifugation, cytoplasmic (C) and nuclear (N) fractions were prepared and 40 μg protein in the C and an equal volume in the N fraction were analyzed by western blotting.

Although several isoforms of USP7 have been predicted (http://www.ncbi.nlm.nih.gov/IEB/Research/Acembly/av.cgi?db=human&q=USP7), with a major variation at the N terminus of the protein (Figure 3A), we found that only one of these isoforms (USP7S) contains a Ser18 phosphorylation site and that this region is highly conserved in different species (Figure S1A). Surprisingly, we found that USP7S detected by our antibodies is not the major USP7 isoform expressed in human cells. When we immunoprecipitated the protein from whole-cell extract using our antibodies we observed a very efficient precipitation of USP7S, although using a commercially available antibody that cross-reacts with all USP7 isoforms (further referred as USP7_total_), we found that USP7S represented only a small proportion of the total USP7 pool (Figure 3B). We also found that this isoform is present in both the nucleus and the cytoplasm (Figure 3C), although its concentration was higher in nucleus. However, taking into account the higher volume of the cytoplasm compared to the nucleus, the total amount of cytoplasmic form of USP7S isoform was calculated at approximately 80%, which was confirmed by western blot analysis of fractionated cell extracts (Figure 3D).

To demonstrate the major role of USP7S in the regulation of Mdm2, we compared the effect of siRNA knockdown of all USP7 isoforms (USP7_total_ siRNA) to a knockdown of this specific isoform. As expected, a knockdown of USP7_total_ resulted in a reduction of all the isoforms of USP7, including USP7S, and consequently led to an increased degradation of Mdm2 (Figure 3E, lane 2). Surprisingly, a knockdown of the USP7S isoform (although only approximately 50% efficient due to the close proximity of the siRNA targeting sequence to the 5′ end of the mRNA) similarly reduced the level of Mdm2 (Figure 3E, lane 3), suggesting that USP7S plays a major role in modulating the cellular levels of Mdm2. The major decrease in Mdm2 levels occurred in the cytoplasm (Figure 3F, compare lanes 1 and 3) and was accompanied by p53 stabilization and accumulation in the nucleus (Figure 3F, compare lanes 2 and 4).

### PPM1G Is the Major Human Phosphatase Involved in Dephosphorylation of USP7S

**Figure 4:**
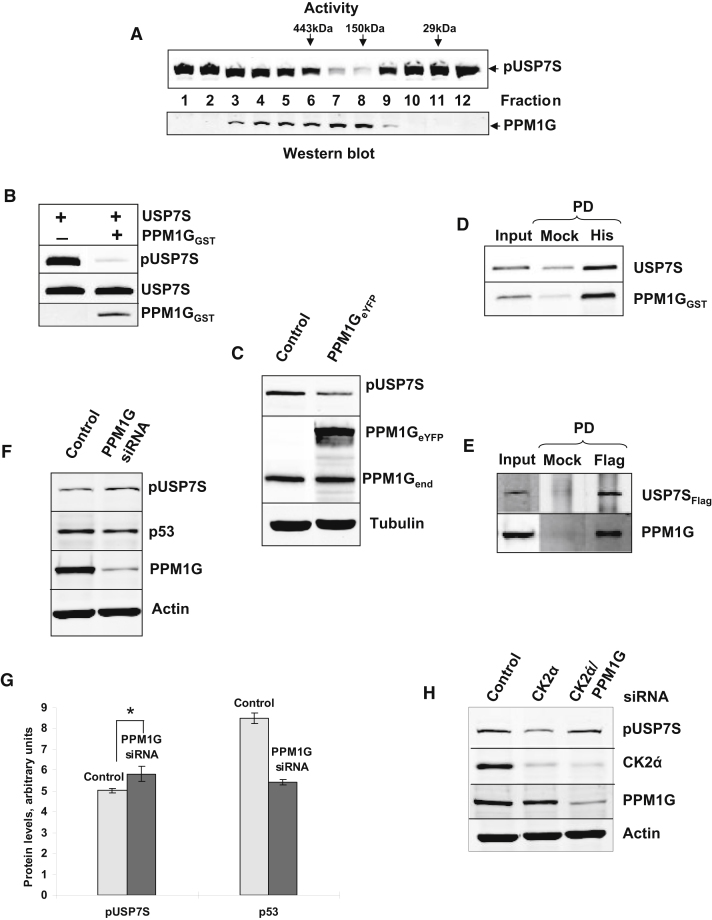
Purification and Identification of PPM1G as the Major Cellular Protein Phosphatase Dephosphorylating USP7S (A) In vitro dephosphorylation of recombinant pUSP7S (1 pmol) by Superdex-200 chromatography fractions purified from HeLa cells analyzed by western blotting using pUSP7S (top panel) and PPM1G antibodies (lower panel) to demonstrate a correlation between PPM1G protein and pUSP7S in vitro dephosphorylation activity. (B) In vitro dephosphorylation of recombinant pUSP7S (2.8 pmol) by recombinant PPM1G (2.8 pmol) produced using an in vitro Wheat Germ Expression System was performed at 30°C for 30 min and analyzed by western blotting. Substrate equal loading and the absence or presence of the phosphatase in the reaction mixture was demonstrated using USP7S and PPM1G antibodies, respectively. (C) HeLa cells were treated with Lipofectamine transfection reagent in the absence (Control) or presence of PPM1G_eYFP_-expressing plasmid (1 pmol) for 24 hr. Cell extracts were prepared and analyzed by western blotting using pUSP7S and PPM1G antibodies. (D) Equal amounts (0.42 pmol) of purified recombinant His-tagged USP7S and PPM1G were incubated at room temperature for 30 min and USP7S was then pulled down using either magnetic beads as a control (Mock PD, 100%) or using magnetic Ni-NTA agarose beads (His PD, 100%), the beads were boiled for 5 min, and proteins were separated by 10% SDS-PAGE and analyzed by western blotting with USP7S and PPM1G antibodies (10% of the input is loaded). (E) AG06173 cells were treated with Lipofectamine RNAiMAX transfection reagent in the presence of both USP7S and PPM1G (0.5 pmol each) expression plasmids for 24 hr. Whole cell extract (500 μg) was used to pull down Flag-tagged USP7S using either magnetic beads as a control (Mock PD, 100%) or Flag-agarose beads (Flag PD, 100%), and the pulldowns were analyzed with USP7S and PPM1G antibodies (15% of the Input is loaded). (F) HCT116 p53^+/+^ cells were treated with Lipofectamine RNAiMAX transfection reagent in the absence (Control) and presence of PPM1G-specific siRNA (200 pmol) for 72 hr. Cells were pelleted by centrifugation, and whole-cell extracts were prepared and analyzed by western blotting with the antibodies indicated. (G) Graphical analysis of the data presented in Figure 4F. pUSP7S protein level was 1.2-fold higher and p53 levels were 1.6-fold lower after PPM1G knockdown compared to the Control, the statistical data are presented as a mean ± SD, ^∗^p < 0.01 as analyzed by Student's t test from three independent experiments. (H) HCT116 p53^+/+^ cells were treated with Lipofectamine RNAiMAX transfection reagent in the absence (Control) and the presence of CK2α′ siRNA (200 pmol) for 48 hr. So that a double knock down could be achieved, CK2α′ siRNA-treated cells were transfected with PPM1G siRNA (200 pmol) for a further 48 hr as described above. Cells were pelleted by centrifugation, and whole-cell extracts were prepared and analyzed by western blotting. See also Figure S2 and Table S2.

Having established that phosphorylation is required for USP7S stability and that dephosphorylation of USP7S leads to its proteasomal degradation, we hypothesized that regulation of USP7S phosphorylation plays a major role in controlling Mdm2 and p53 in the cellular response to DNA damage. We thus pursued purification of a human phosphatase responsible for USP7S dephosphorylation at Ser18. Using recombinant USP7S purified from insect cells (this protein is phosphorylated and referred to as pUSP7S) as a substrate in an in vitro dephosphorylation reaction, we fractionated HeLa whole cell extracts over a series of chromatographic columns (Figure 4A and Figures S2A–S2D). When fractions from the final hydroxyapatite column containing pUSP7S-specific phosphatase activity (Figure S2D) were analyzed by mass spectrometry, the serine/threonine phosphatase PPM1G (also known as PP2Cγ) was found as the sole phosphatase in these fractions (Table S2). We next used purified recombinant human PPM1G protein produced by an in vitro Wheat Germ Expression System and demonstrated its ability to dephosphorylate pUSP7S in vitro (Figure 4B). Dephosphorylation of USP7S by PPM1G is specific as we did not observe pUSP7S dephosphorylation by the phosphatase PTEN, which is also involved in the DNA damage response (Figure S2E). To confirm that PPM1G can dephosphorylate USP7S in living cells, we overexpressed this protein in HeLa cells and demonstrated that elevated levels of PPM1G led to dephosphorylation of endogenous USP7S (Figure 4C). In contrast, overexpression of the DNA damage inducible phosphatase Wip1 did not affect USP7S phosphorylation (Figure S2F). Comprehensive proteomic analysis of deubiquitylating enzymes and their interacting partners previously indicated that USP7 may possibly interact with PPM1G (Sowa et al., 2009). In agreement with this, we found that the PPM1G protein efficiently interacts with USP7S both in vitro and in vivo (Figures 4D and 4E).

To examine the physiological consequences of PPM1G loss of function in human cells, we knocked down PPM1G in HCT116 p53^+/+^ cells. This resulted in a moderate increase in USP7S phosphorylation (Figures 4F and 4G), supporting our earlier finding that the majority of USP7S is already phosphorylated (Figure 1B). However, the observed increase in USP7S phosphorylation was sufficient to moderately downregulate p53 (Figures 4F and 4G). To further demonstrate that the phosphorylation status of USP7S depends on the balance between CK2 and PPM1G cellular activities, we knocked down either or both proteins using siRNA. A knockdown of CK2 resulted in reduced USP7S phosphorylation, however a simultaneous knock down of both CK2 and PPM1G restored the levels of phosphorylated USP7S (Figure 4H). This demonstrates that CK2 and PPM1G have counteracting activities in regulating the phosphorylation status of USP7S.

### DNA Damage Leads to ATM-Dependent USP7S Dephosphorylation by PPM1G

**Figure 5:**
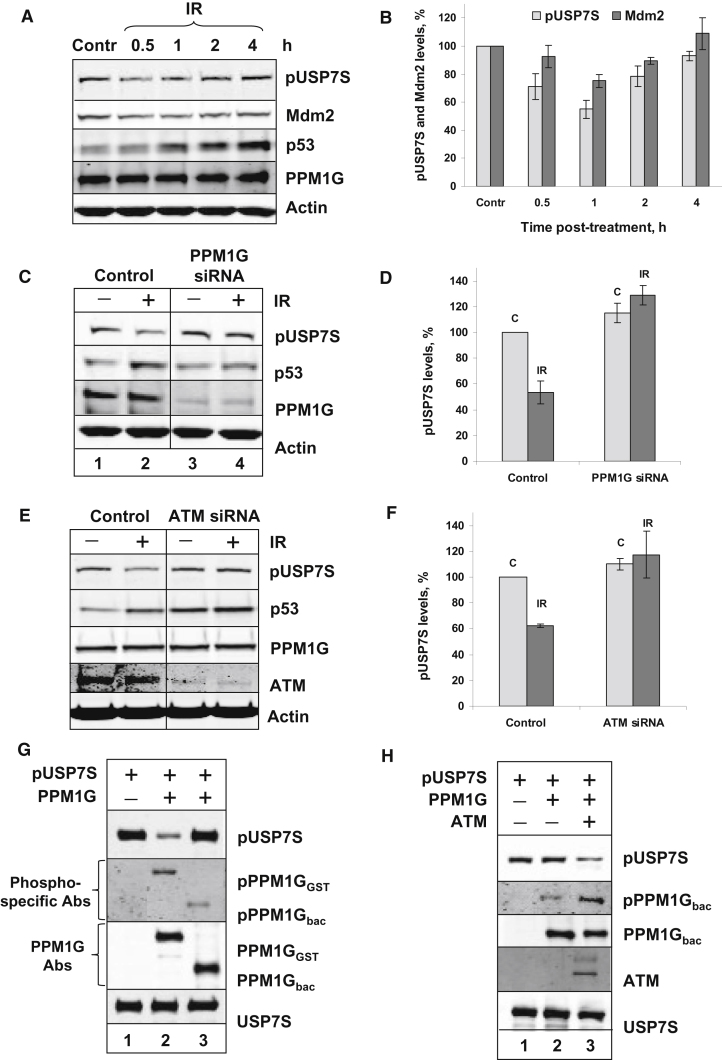
ATM-Dependent Dephosphorylation of USP7S by PPM1G in Response to DNA Damage (A) AG06173 cells were either untreated (Contr) or exposed to 10 Gy ionizing radiation (IR) and harvested at the indicated time points after treatment. Cells were pelleted by centrifugation, and whole-cell extracts were prepared and analyzed by western blotting. (B) Graphical analysis of the data presented in (A). Statistical data are presented as a mean ± SD of three independent biological experiments. (C and E) AG06173 cells were treated with Lipofectamine RNAiMAX transfection reagent in the absence (Control) or presence of PPM1G or ATM siRNA (200 pmol of each) for 72 hr. Cells were harvested either without any additional treatment (–) or 1 hr after treatment with 10 Gy ionizing radiation (+). Whole-cell extracts were prepared and analyzed by western blotting. (D and F) Graphical analysis of the data presented in (C) and (E), respectively. Statistical data are presented as a mean ± SD of three to five independent biological experiments. (G) In vitro dephosphorylation of recombinant pUSP7S (2.8 pmol) by recombinant PPM1G (2.8 pmol) which was prepared either using in vitro Wheat Germ Expression System in mammalian cells (PPM1G_GST_) or overexpressed and purified from bacterial cells (PPM1G_bac_). The reactions were performed at 30°C for 30 min and analyzed by western blotting. The level of PPM1G phosphorylation was assessed using antibodies specific to ATM/R phosphorylated SQ/TQ motif. Substrate equal loading and, the absence or presence of the phosphatase in the reaction mixture, were demonstrated using USP7S and PPM1G antibodies, respectively. (H) In vitro dephosphorylation of recombinant pUSP7S (2.8 pmol) by recombinant PPM1G purified from bacteria (2.8 pmol) which was phosphorylated by ATM in vitro prior to the reactions. The reactions were performed and analyzed as described above. See also Figure S3.

Intriguingly, since USP7S is predominantly phosphorylated in human cells (Figure 1B), this suggests that PPM1G, which is present in unstressed cells, does not continually dephosphorylate USP7S implying that the cellular activity of PPM1G, and correspondingly the phosphorylation status of USP7S, may be regulated by DNA damage. To examine this, we treated primary fibroblasts with 10 Gy ionizing radiation and analyzed the DNA damage stress response for several hours after irradiation. We found that 30–60 min after irradiation, the level of USP7S phosphorylation was decreased by approximately 40%–50%, which was followed by a decrease in Mdm2 and an increase in p53 levels (Figures 5A and 5B). Similar data were obtained with HCT116 p53^+/+^ colon cancer cells (Figure S3A). To demonstrate that USP7S dephosphorylation in response to irradiation is dependent on PPM1G, we irradiated cells after PPM1G knockdown and under such conditions did not observe any USP7S dephosphorylation in response to DNA damage (Figures 5C and 5D). As expected, p53 levels, which were significantly upregulated in response to irradiation (Figure 5C, lane 2), did not increase in response to irradiation after PPM1G depletion (Figure 5C, lane 4). These data further support an important role for PPM1G in the induction of p53 in response to DNA damage. Similar data were also obtained with HCT116 p53^+/+^ colon cancer cells (Figure S3B). Since ATM regulates the majority of the cellular DNA damage responses and PPM1G was previously suggested as a potential target for ATM phosphorylation (Matsuoka et al., 2007), we tested whether USP7S dephosphorylation by PPM1G is ATM-dependent. To this end, we performed a knockdown of ATM and demonstrated that this abolished USP7S dephosphorylation in response to irradiation (Figures 5E and 5F), suggesting that USP7S dephosphorylation by PPM1G is ATM-dependent. However, we were unable to monitor the corresponding p53 response since depletion of ATM itself led to elevated p53 levels, that were not significantly further increased in response to irradiation (Figure 5E). Using recombinant proteins, we have further confirmed that ATM phosphorylation is required for PPM1G activation. First, we demonstrated that human PPM1G purified from eukaryotic cells (PPM1G_GST_) is active in an in vitro dephosphorylation of pUSP7S (Figure 5G, lane 2). Using antibodies that were shown to recognize phosphorylated SQ/TQ motifs (consensus for ATM phosphorylation sites), we were able to show that PPM1G_GST_ is partially phosphorylated at ATM-specific sites (Matsuoka et al., 2007) (Figure 5G, lane 2). In contrast, unphosphorylated human PPM1G purified from bacterial cells (PPM1G_bac_) shows no pUSP7S dephosphorylation activity (Figure 5G, lane 3) and only weakly cross-reacted with the SQ/TQ phosphospecific antibodies. Second, we phosphorylated PPM1G_bac_ with recombinant ATM protein and demonstrated that phosphorylation activates PPM1G_bac_ activity (Figure 5H, compare lanes 2 and 3). Cumulatively, this suggests that DNA damage induced ATM-dependent phosphorylation of PPM1G is required for its activation and subsequent dephosphorylation of pUSP7S.

### PPM1G Depletion Results in Accumulation of Unrepaired DNA Damage and p53-Independent Cell-Cycle Arrest

**Figure 6:**
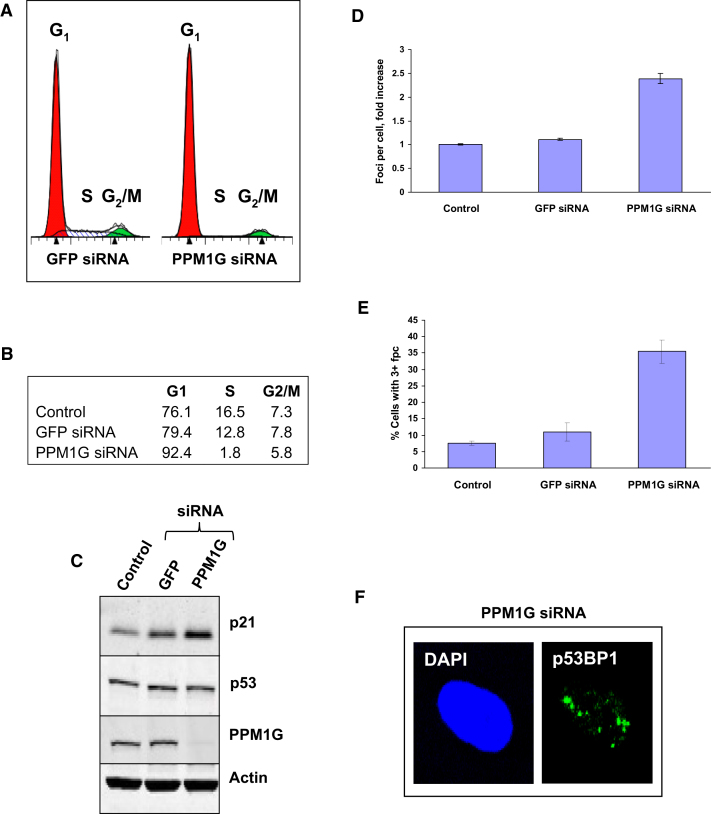
PPM1G Depletion Results in Accumulation of p53BP1 Foci and p53-Independent Cell-Cycle Arrest (A–C) AG06173 cells were either left untreated (Control) or treated with Lipofectamine RNAiMAX transfection reagent in the presence of GFP or PPM1G siRNA (200 pmol) for 48 hr. In (A) and (B), cells were collected by trypsinisation and subjected to FACS analysis. Cell-cycle profiles and quantification of cell cycle distribution are shown, respectively. In (C) cells were pelleted by centrifugation, whole-cell extracts were prepared and analyzed by western blotting. (D–F) AG06173 cells were grown on coverslips for 24 hr to 30%–50% confluency, left untreated (Control) or treated in the presence of GFP or PPM1G specific siRNA as described above and then immunostained with p53BP1 antibodies followed by Alexa Fluor 488 anti-rabbit IgG. Detection of p53BP1 foci formation was performed, the obtained data were analyzed and presented as the number of foci per cell normalized to Control (D) and as the percentage of cells containing three or more foci per nucleus (E). Statistical data are presented as a mean ± SD of three to five independent biological experiments. An example of p53BP1 foci formation in PPM1G-depleted AG06173 cells is shown (F).

To assess the biological importance of USP7S regulation by PPM1G, we examined the consequences of PPM1G depletion in AG06173 human primary fibroblasts. We found that a knockdown of PPM1G resulted in a cell-cycle delay, which occurs in the G1 phase (Figure 6A and B). As expected, p53 levels did not change (Figure 6C), since the DNA damage signal transduction pathway was disrupted by PPM1G depletion and, as a result cells behaved similar to p53-deficient cells. We therefore observed p53-independent induction of p21 protein that was followed by cell-cycle arrest in G1 phase, as previously described (Aliouat-Denis et al., 2005). Interestingly, cells that arrest in G1 phase after PPM1G knockdown also accumulate unrepaired DNA strand breaks, as detected by immunostaining with p53BP1 antibodies (Figures 6D–6F). This suggests that cells deficient in PPM1G are unable to efficiently repair endogenous DNA damage and highlights an important role for PPM1G in the cellular response to DNA damage.

## Discussion

### Phosphorylated USP7 Isoform Plays a Major Role in Mdm2 and p53 Regulation

Although several different isoforms of USP7 have been reported, very little is known about their biological role. Fortunately, in previous studies that have established the role of USP7 in controlling Mdm2 and p53 protein levels, the specific Ser18-containing USP7 isoform protein (USP7S) and corresponding expression plasmids were used, though without prior knowledge that this is not the major USP7 isoform (Brooks et al., 2007; Li et al., 2004; Meulmeester et al., 2005). Our data clearly demonstrate that regulation of Mdm2 and p53 cellular levels is accomplished by this specific isoform of USP7, whose stability and ability to regulate Mdm2 levels are preserved by phosphorylation of serine 18. We found that the majority (60%–80%) of USP7S is phosphorylated in human cells and although its concentration is higher in the nucleus, the bulk of the enzyme is present in the cytoplasm. We also demonstrated that phosphorylated USP7S plays a key role in the regulation of Mdm2 stability, and thus controls degradation of p53. These findings have raised a question of the biological role of other USP7 isoforms, which are most probably involved in regulation of different pathways of cellular responses to stress, and should be the major focus of future studies.

### USP7S Phosphorylation by CK2

The CK2 protein kinase is a highly conserved, ubiquitous and constitutively active serine/threonine kinase found in both the cytoplasm and nucleus, where it phosphorylates many proteins involved in different cellular processes, including those involved in the DNA damage response (Ahmed et al., 2002; Meggio and Pinna, 2003). CK2 was reported to phosphorylate p53 (Meek et al., 1990), Mdm2 (Guerra et al., 1997), XRCC1 (Loizou et al., 2004; Parsons et al., 2010), and APE1 (Fritz and Kaina, 1999). In several cases, it was demonstrated that phosphorylation by CK2 regulates protein stability by preventing their ubiquitylation and consequent proteasomal degradation (Parsons et al., 2010; Torres et al., 2003). Although, the phosphorylation of USP7S at Ser18 in living cells was previously reported, the protein kinase involved was not identified and the biological role of this phosphorylation remained unknown (Fernández-Montalván et al., 2007). We have now demonstrated that USP7S is phosphorylated by CK2 both in cells and in an in vitro reaction reconstituted with recombinant proteins. Since we found that up to 80% of USP7S is phosphorylated and that dephosphorylation by PPM1G is activated only in response to DNA damage, we propose that phosphorylation is essential for USP7S stability and consequently for the downregulation of p53 protein levels in unstressed cells.

### The Role of PPM1G in the DNA Damage Response

**Figure 7:**
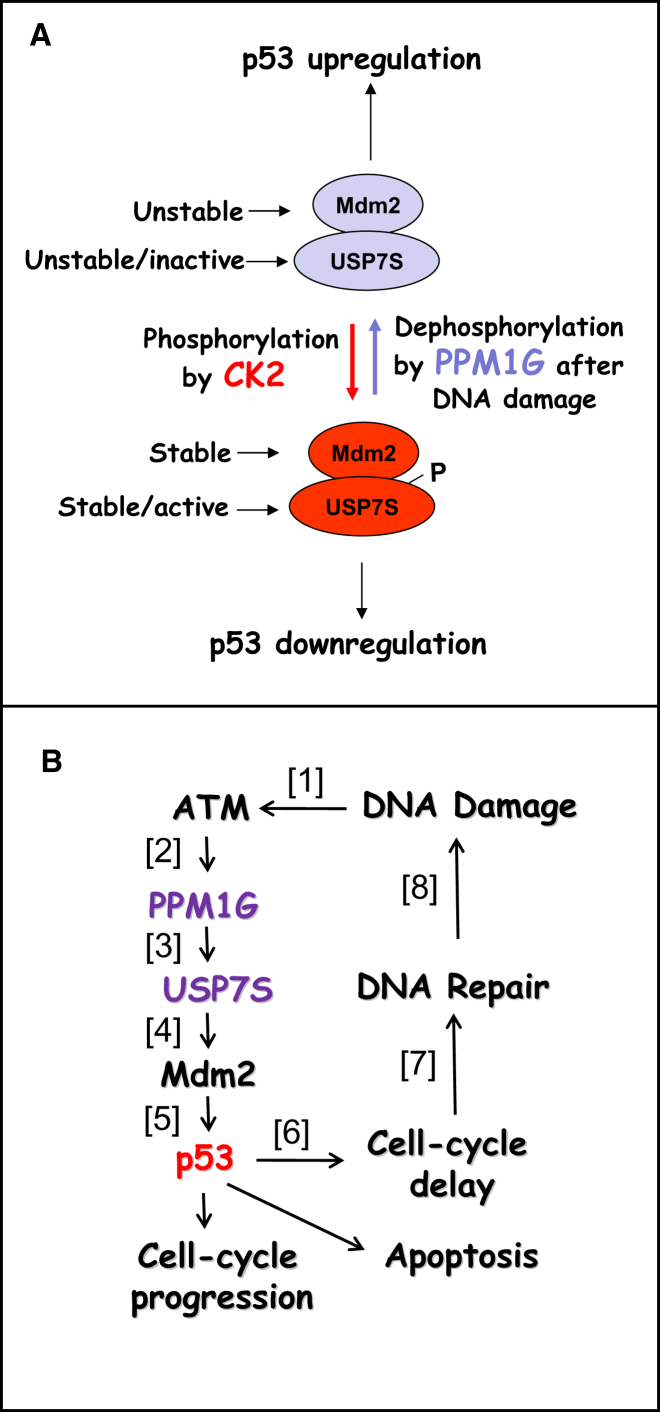
Proposed Models for Coordination of DNA Repair and Cell-Cycle Progression by Modulating USP7S Phosphorylation Status and Regulation of p53 Stability by USP7S (A) In unstressed cells, the majority of USP7S is phosphorylated by CK2 and active in deubiquitylation and stabilization of Mdm2 that in turn leads to p53 ubiquitylation and proteasomal degradation. If DNA damage is detected, USP7S is dephosphorylated by PPM1G and degraded, thus inducing Mdm2 degradation and consequent p53 stabilization. (B) DNA damage activated ATM [1] stimulates PPM1G activity [2] that dephosphorylates USP7S [3], thus promoting its inactivation and degradation, consequently leading to Mdm2 self-ubiquitylation and proteasomal degradation [4] and p53 stabilization [5]. If the DNA damage signal is not sufficient enough, cells progress through to replication. Otherwise at this stage, p53 levels are increased proportionally to the DNA damage signal that allows an adequate delay in cell-cycle progression [6] to accomplish DNA repair [7]. This cycle yields a new level of DNA damage [8] and will be repeated until cells will be allowed to undergo replication or will be redirected to apoptosis in the case of excessive DNA damage.

Protein phosphorylation/dephosphorylation is an important mechanism that regulates many cellular processes, including DNA replication and repair. Frequently phosphorylation controls protein ubiquitylation and thus regulates the protein lifetime and activity (Moorhead et al., 2007; Wimmer et al., 2008). It is well established that downregulation of the DNA damage response is accomplished by the protein phosphatase PPM1D (also known as Wip1) that is induced at the latter stages of this process where it dephosphorylates ATM, Mdm2 and p53 (Lu et al., 2008). However, the phosphatase involved in the activation of the p53-dependent DNA damage response has not been recognized. In this study, we identified PPM1G as the major phosphatase involved in USP7S dephosphorylation in response to DNA damage. It was previously suggested that PPM1G interacts with USP7, however the biological role of this interaction was not clear (Sowa et al., 2009). We have now shown that when DNA damage is detected, PPM1G is activated and dephosphorylates USP7S that leads to its degradation and consequently to Mdm2 degradation and accumulation of p53 (Figure 7A). Although PPM1G was originally identified as a factor involved in pre-mRNA splicing, it was later implicated in DNA replication control (Suh et al., 2006) and the DNA damage response (Kimura et al., 2006), however the precise role of PPM1G in these processes remained obscure. It was also demonstrated that overexpression of PPM1G induces cell-cycle arrest (Suh et al., 2006) and that chicken DT-40 cells deficient in PPM1G have increased sensitivity to irradiation (Kimura et al., 2006), and these are now both in line with our findings that PPM1G is required for activation of the DNA damage response. Although the exact mechanism of PPM1G activation after irradiation was previously unclear, PPM1G was recently identified as a target for ATM in a genome wide screening for ATM-dependent protein phosphorylation induced by ionizing radiation (Matsuoka et al., 2007). Our data demonstrate that ATM phosphorylation regulates PPM1G activity and that ATM knockdown abolishes DNA damage dependent dephosphorylation of USP7S by PPM1G, supporting our model where PPM1G is activated by direct ATM phosphorylation.

### PPM1G Regulation of p53 Levels in Response to DNA Damage

Historically p53 is considered as the major player in the cellular response to exogenous DNA damage stress, such as UV or ionizing irradiation (Vousden and Lane, 2007). However, it is clear that it is also a major part of the routine DNA quality control prior to every DNA replication event, since “spontaneous” or endogenous DNA lesions are constantly arising as a result of the chemical instability of the DNA molecule and DNA damage is produced by endogenous mutagens, such as reactive oxygen species (Lindahl, 1993). However, since DNA repair systems continually remove DNA lesions, the delicate balance between these counteracting processes results in p53 cellular levels that are not stable but are continually changing through attenuated pulses reflecting cellular DNA damage and repair dynamics (Loewer et al., 2010). The misbalance between DNA damage and repair results in higher cellular p53 levels and is found elevated in precancerous cells indicating an increased DNA damage load or deficient DNA repair (Bartkova et al., 2005).

Although the “classical” model for the DNA damage stress response is based on phosphorylation induced p53 stabilization and its activation as a transcription factor, recent data clearly indicated that upregulation of p53 in response to excessive DNA damage is mainly controlled by Mdm2 (de Rozieres et al., 2000; Kruse and Gu, 2009; Ringshausen et al., 2006; Vousden and Lane, 2007; Vousden and Prives, 2009). On the other hand, Mdm2 levels are regulated by the USP7 protein that prevents Mdm2 self-ubiquitylation and proteasomal degradation (Cummins and Vogelstein, 2004). The key question that remains to be answered is how the signal from DNA damage is quantitatively transmitted to Mdm2 and USP7 proteins to up- or downregulate p53 levels. In this study, we discovered a pathway (Figure 7B) in which, in response to DNA damage, ATM-dependent dephosphorylation of a specific isoform of USP7 (USP7S) by the PPM1G protein phosphatase downregulates its activity and stability and thus downregulates Mdm2 levels and consequently adjusts p53 levels to the amount of DNA damage. We propose that the strength of the signal (p53 level) allows cells to undergo DNA replication or to delay cell cycle progression exactly for the time needed to complete DNA repair or to undergo apoptosis, if the DNA damage is too excessive (Figure 7B). In the case of a PPM1G deficiency, cells behave similar to p53-deficient cells, since DNA damage signal transduction is disrupted and cells enter DNA replication without a cell-cycle delay for repair, thus accumulating DNA damage. However, as previously described, cells have a backup mechanism that leads to p53-independent, but DNA damage-dependent, accumulation of p21 that results in restrictions in further replication and a G1 cell-cycle arrest (Aliouat-Denis et al., 2005). Indeed, as we demonstrated, replication without coordination of DNA repair and replication (when PPM1G is downregulated) leads to accumulation of DNA strand breaks, p21 induction and finally results in cell-cycle arrest at the G1 phase (Figure 6).

In conclusion, our findings provide a molecular mechanism for DNA damage dependent regulation of cellular p53 levels, and coordination of the DNA damage response at the cellular level, by regulation of USP7 phosphorylation. This is a previously unknown DNA damage signal transduction pathway that links ATM and p53, and the proteins involved in this pathway may be important targets for cancer therapy.

## Experimental Procedures

Information regarding antibodies, plasmids, proteins, whole-cell extracts preparation and cell fractionation, western blotting, mass spectrometry analysis, immunofluorescence, real-time PCR, FACS analysis, and detection of foci formation can be found in Supplemental Experimental Procedures.

### Whole-Cell Extracts and Cell Fractionation

Whole-cell extracts were prepared by Tanaka's method (Tanaka et al., 1992) and cell fractionation was performed as recently described (Woodhouse et al., 2008).

### In Vitro Kinase and Phosphatase Assays

For preparation of a substrate for the in vitro kinase assay, recombinant USP7S (100 pmol) was dephosphorylated in the presence of shrimp alkaline phosphatase (50 U) in buffer containing 20 mM Tris-HCl (pH 8.0), 10 mM MgCl_2_ for 1 hr at 37°C with shaking. The reaction mixture was made up to 0.5 M NaCl and 5 mM Imidazole and applied to a 1 ml HisTrap HP column (GEHealthcare). Dephosphorylated USP7S was eluted with 250 mM imidazole-containing buffer, dialyzed against buffer containing 50 mM Tris-HCl (pH 8.0), 50 mM KCl, 1 mM EDTA, 10% glycerol, concentrated and stored at −80°C until required. The efficiency of dephosphorylation was checked by western blotting with polyclonal antibodies raised against USP7S phosphorylated at Ser18 (pUSP7S). Recombinant dephosphorylated USP7S (1 pmol) was incubated with fractions purified from HeLa whole cell extract in buffer containing 20 mM Tris-HCl (pH 7.4), 50 mM KCl, 10 mM MgCl_2_, 1 mM DTT, 5% glycerol, 1 mM ATP and 1% (v/v) of phosphatase inhibitor cocktail I for 30–60 min at 30°C with shaking. For stopping the reaction, SDS-PAGE sample buffer (25 mM Tris-HCl [pH 6.8], 2.5% β-mercaptoethanol, 1% SDS, 5% glycerol, 1 mM EDTA, 0.15 mg/ml bromophenol blue) was added to the samples, which were then heated for 5 min at 95°C prior to separation of the proteins on a 10% SDS-polyacrylamide gel, followed by transfer to a PVDF membrane and immunobloting with pUSP7S antibodies. For the in vitro phosphatase assay, recombinant USP7S (1 pmol) was incubated with fractions purified from HeLa whole-cell extract or recombinant PPM1G purified either from bacterial cells or produced by an in vitro Wheat Germ Expression System (Abnova) in buffer containing 50 mM Tris-HCl (pH 7.4), 10 mM MgCl_2_, 0.2 mM EGTA, 0.1% (v/v) β-mercaptoethanol, 100 μg/ml BSA, and 10 μM each of staurosporin and 4,5,6,7-tetrabromo-1H-benzotriazole (TBB) for 30–60 min at 30°C with shaking. Alternatively, recombinant PPM1G purified from bacteria (3 pmol) was incubated with purified recombinant ATM (kindly provided by T. Paull) in buffer containing 50 mM HEPES (pH 7.5), 50 mM KCl, 10 mM MgCl_2_, 10 mM MnCl_2_,1 mM ATP, 1 mM DTT, and 5% glycerol for 90 min at 30°C with shaking or treated in the same conditions with no ATM added and then used in the in vitro dephosphorylation. Dephosphorylation reactions were performed and analyzed as described above.

### Purification of USP7S-Specific Kinase and Phosphatase Activities

Whole-cell extract from 20 g HeLa cells pellet was prepared by Tanaka`s method and dialysed against Buffer A (50 mM Tris-HCl [pH 8.0], 1 mM EDTA, 5% glycerol, 1 mM DTT, and 0.1 mM PMSF) containing 150 mM KCl. The extract was applied to a phosphocellulose column, the flow-through (PC150 fraction) was collected and proteins bound to the column were eluted in Buffer A containing 1 M KCl (PC1000 fraction). PC1000 containing USP7S kinase activity was dialysed against Buffer A containing 50 mM KCl and further fractionated on a 5 ml HiTrapQ HP column (GE Healthcare) with a linear gradient of 50 mM-1 M KCl. Active fractions were pooled, concentrated using Amicon Ultra-10 filter units (Millipore) and separated a Superdex 200 HR 10/30 column (GE Healthcare) in Buffer A containing 150 mM KCl. Active fractions were pooled and loaded onto a 1 ml MonoQ column (GE Healthcare) in Buffer A containing 150 mM KCl. Proteins were eluted using a linear gradient of 150–700 mM KCl. Active fractions were identified, pooled, dialysed against buffer containing 5 mM potassium phosphate (pH 7.0), 5% glycerol and applied onto a 1 ml hydroxyapatite column (GE Healthcare). The flow-through was collected and proteins were eluted using a linear gradient 5 500 mM phosphate. At each purification step, aliquots of the fractions obtained were analyzed for USP7S kinase activity using dephosphorylated USP7S as a substrate as described above. Fractions identified as active were pooled for the next chromatography step.

Similar purification scheme was applied for isolation of USP7S specific phosphatase activity excluding the fractionation on 1 ml MonoQ column. Fractions were analyzed for USP7S dephosphorylation activity using phosphorylated USP7S as a substrate as described above.

### RNA Interference

Cells were transfected with Lipofectamine RNAiMAX reagent (Invitrogen) according to the manufacturer's protocol. The following siRNA sequences were used: 5′-GAUGACUACCAGCUGGUUC-3′ for CK2α (Zhang et al., 2007), 5′ AAAGCUGCGACUGAUAGAUUG-3′ for CK2α′ (Luo et al., 2004), 5′ AGGCUACCAUGACUAUUGA-3′ for PPM1G (Petri et al., 2007), and 5′-AACATACTACTCAAAGACATT-3′ for ATM (Andreassen et al., 2004). For knockdown of all isoforms of USP7 a mixture of two sequences (5′-ACCCUUGGACAAUAUUCCU-3′ and 5′-AGUCGUUCAGUCGUCGUAU-3′) was used (van der Horst et al., 2006). For knockdown of Ser18-containing USP7 isoform, 5′-AGCGGGCGAGCAGCAGUUG-3′ sequence was used. The GFP siRNA sequence (5′-GCUGACCCUGAAGUUCAUCUU-3′) served as a control.

### Irradiation

Cells were subjected to ionizing radiation using GSR-D1 137Cs γ-irradiator (RPS Services Limited) at a dose rate of 1.8 Gy/min (10 Gy dose).

### Statistical Analysis

Results are presented as a mean ± standard deviation. Data of at least three to five independent biological experiments were used. Statistical analysis was performed using Student's paired t test with a two-tailed distribution.

## Supplemental Information

Document S1. Supplemental Experimental Procedures, Figures S1–S3, and Tables S1 and S2
