# Supplementary material for: ATM-Dependent Downregulation of USP7/HAUSP by PPM1G Activates p53 Response to DNA Damage
Source: Mol Cell. 2012 Mar 30;45(6):801–13. doi: 10.1016/j.molcel.2012.01.021 (PMC3401373; doi:10.1016/j.molcel.2012.01.021)
Supplement: Document S1. Supplemental Experimental Procedures, Figures S1–S3, and Tables S1 and S2 [file mmc1.pdf]

**Molecular Cell,** ***Volume 45***

# Article

# ATM-Dependent Downregulation of USP7/HAUSP by PPM1G Activates p53 response to DNA damage

**Svetlana V. Khoronenkova, Irina I. Dianova, Nicola Ternette, Benedikt M. Kessler, Jason L. Parsons, and Grigory L. Dianov**

## Supplemental Experimental Procedures

**Antibodies, proteins and inhibitors.** USP7 antibodies purchased from Bethyl Laboratories ($USP7_{total}$) were generated against a synthetic peptide corresponding to amino acids 20-50 of human USP7. Mdm2 antibodies were purchased from AbD Serotec, CK2α, CK2α', ATM, p21 and actin antibodies were from Abcam, PTEN antibodies were from Santa Cruz, tubulin and fibrillarin antibodies were purchased from Sigma-Aldrich, FLAG antibodies were purchased from Agilent Technologies, 53BP1 and phosphorylated S1423 BRCA1 antibodies were from Bethyl Laboratories, PPM1G antibodies were kindly provided by O. Gruss. Polyclonal USP7 antibodies against both the peptide EQQLpSEPEDMEMEAGDT phosphorylated at serine 18 (pUSP7S) and the similar but non-phosphorylated peptide (USP7S; amino acids 14-30 of USP7 protein sequence) were produced by Biomatik. Ubiquitin, E1 and E2 enzymes were purchased from Boston Biochemicals. Mdm2 and $PPM1G_{GST}$ were from Abnova. Casein kinase 2 was purchased from New England Biolabs and shrimp alkaline phosphatase was from USB Corporation. $PPM1G_{bac}$, PTEN and Wip1 were expressed in *E. coli* cells and purified using HisTrap HP column chromatography (GE Healthcare). ATM was kindly provided by T. Paull and R. Gatti. Phosphatase inhibitor cocktail I, staurosporin, specific CK2 inhibitor 4,5,6,7-tetrabromo-1H-benzotriazole (TBB) and all protease inhibitors were purchased from Sigma-Aldrich. The level of PPM1G phosphorylation was assessed using antibodies pS1423 BRCT1 specific to ATM/R phosphorylated SQ/TQ motif.

**Plasmids.** *Mdm2*, *p53* and *Wip1* containing vectors for mammalian expression were kindly provided by Drs. K. Vousden, E. Hammond and D. Bulavin respectively. YFP-tagged *PPM1G* mammalian expression vector was kindly provided by Dr. O. Gruss. The *PPM1G* gene was sub-cloned into pCMV-3Tag3a vector containing C-terminal 3 × Flag tag (Agilent Technologies) for mammalian expression using ligation-independent cloning (LIC) technique (Aslanidis and de Jong, 1990). Mammalian expression vector encoding the *usp7s* gene containing an N-terminal Flag-tag was purchased from Addgene (Addgene plasmid 16655). This plasmid was also used both to introduce a single point mutation Ser18Ala (USP7S18A) using the QuikChange® Site-Directed Mutagenesis Kit from Agilent Technologies and to prepare USP7ΔN isoform containing plasmid for mammalian expression (GenBank Accession Number AK302771) using LIC technique. Mutant *usp7S18A* gene was cloned into pFastBac-LIC-Bse vector containing a N-

terminal His-tag (kindly provided by P. Savitsky, SGC Oxford, UK) using LIC (Aslanidis and de Jong, 1990). The vector was then used to generate recombinant baculovirus using the Bac-to-Bac system (Invitrogen). Ready-to-use baculovirus containing His-tagged USP7S was kindly provided by R. Everett.

**Expression and purification of USP7S and USP7S18A.** To express wild-type USP7S and mutant USP7S18A, *Sf9* insect cells were grown in suspension in Sf-900 II SFM medium (Invitrogen) supplemented to a final concentration of 2 % (v/v) foetal bovine serum, 50 U/mL of penicillin and 50 μg/mL of streptomycin. 400 mL of *Sf9* cells (density $2x10^6$ cells/mL) were infected by 12 mL of plaque-purified and amplified virus stock and then incubated at 27 ºC at 150 rpm for 72 h. The cells were harvested at 1500 × g for 15 min, washed in phosphate-buffered saline (PBS) and frozen at -80 ºC until required. Cell pellets were resuspended in 20 mL of buffer containing 20 mM Tris-HCl, 0.5 M NaCl, pH 8.0, 5 % glycerol, 5 mM imidazole, 1 % of Nonidet P-40 and 0.1 mM DTT. To obtain clear cell free extracts, the cells were lysed by sonication on ice, centrifuged at 20000 rpm for 20 min at 4 ºC and the supernatant was filtered through a 1.0 μm filter. His-tagged USP7S and USP7S18A were then purified using a 1 ml HisTrap HP column (GEHealthcare) as recommended by the manufacturer. Proteins were eluted using a linear gradient of 5-250 mM imidazole-containing buffer and fractions containing USP7S were dialysed against buffer containing 50 mM Tris-HCl, pH 8.0, 50 mM KCl, 1 mM EDTA, 10 % glycerol and stored at -80 ºC until required.

**Western Blots.** Western blots were performed by standard procedure as recommended by the vendor (Novex). Blots were visualized and quantified using the Odyssey image analysis system (Li-Cor Biosciences).

**Protein identification and mapping of ubiquitination sites by tandem mass spectrometry.** Proteins in chromatography fractions were precipitated using chloroform and methanol as described previously (Wessel and Flugge, 1984), followed by in-solution trypsin digestion. Digested material was desalted using C18 Sep-Pack cartridges (Waters, Milford, USA) according to the manufacturer's instructions and concentrated by vacuum centrifugation. Coomassie gel bands were excised and subjected to in-gel trypsin digestion as described (Borodovsky et al., 2002). Digested material was analysed by LC-MS/MS tandem mass spectrometry using either a high capacity iontrap (HCTplus™, Bruker Daltonics) as described (Batycka et al., 2006) or a nanoAcquity UPLC system coupled to a quadrupole time-of-flight (QTOFpremier™) tandem mass spectrometer (Waters). The interpretation and presentation of MS/MS data was performed according to published guidelines (Taylor and Goodlett, 2005) MS/MS spectra (peak lists) were searched against the SwissProt database (release version 54.0, 07/2007, number of entries 276256) using Mascot version 2.2 (Matrixscience). In addition, individual MS/MS spectra for peptides with a Mascot Mowse score lower than 40 (Expect <0.015) were inspected manually and included in the statistics only if a series of at least 4 continuous y or b ions were observed. The local "in-house" Mascot server used for this study is supported and maintained by the Computational Biology Research Group at the University of Oxford.

**Real-time PCR.** Total RNA was purified using the RNeasy kit (Qiagen) and cDNA was prepared using the SuperScript RT-PCR system (Invitrogen). $USP7_{total}$ and USP7S transcripts were amplified using the following pairs of primers: 5′-AGGCTCAGAAGCGGAAGG-3′ and 5′-AAACTGGTCCTCTGCGACTATC-3′; 5′-CGAGCAGCAGTTGAGCG-3′ and 5′-CGCTCCACAGTGAACTGAAA-3′; respectively. Expression values obtained were normalised to actin (5′-AGGCACCAGGGCGTGAT-3′ and 5′-CGCCCACATAGGAATCCTTCT-3′). All primers used were optimised for their concentration and the final product size. Quantitative rtPCR was performed using Absolute Blue QPCR SYBR low ROX Mix (Thermo Scientific) according to the manufacturer′s protocol. Reactions were carried out in triplicate for each target transcript using a 7500 Fast Real-Time PCR System (Applied Biosystems). The comparative $C_T$ method was applied for quantification of gene expression. The results were analysed statistically as described in the Experimental Procedures section of the main text and represented as a fold change of mRNA levels.

**Immunofluorescence and foci detection.** Cells were seeded on coverslips in 12-well plates ($1\times10^4$ cells/well) and grown for a further 24 h. Sub-confluent cell monolayers on coverslips were fixed with 4 % paraformaldehyde for 30 min at room temperature, then permeabilized with 0.2 % (w/v) Triton X-100 for 10 min at 4°C and blocked with 2 % (w/v) BSA - Fraction V for 1 h at room temperature, followed by exposure to primary and secondary antibodies. Primary USP7S, pUSP7S, PPM1G and 53BP1 antibodies were as described above. Secondary antibodies were Alexa Fluor 488 $F(ab')_2$ fragment of goat anti-rabbit (Invitrogen). Slides were mounted with Vectashield® medium with 4,6-diamidino-2-phenylindole (DAPI) from Vector Laboratories and imaged using a BioRad MRC 600 confocal microscope and x60 oil objective. The green and red channels used 488 nm (FITC) and 633 nm (Cy5) argon laser lines for excitation. Slides for the detection of 53BP1 foci formation were prepared in duplicate and 100 cells per slide were counted. The statistical analysis of the obtained data was performed as described in the Experimental Procedures section of the main text.The results were normalized to non-treated control sample and represented both as foci per cell values and the percentage of cells with three and more foci.

**FACS analysis.** Harvested cells were fixed in ice-cold 70 % ethanol for at least 30 min. After removal of the fixative solution by centrifugation, cells were resuspended in PBS containing 10 µg/ml propidium iodide (Sigma). Samples were run on a Becton-Dickinson FACScan (BD Biosciences, Oxford, UK) and the obtained data were analysed using Modfit LT software (Verity Software House).

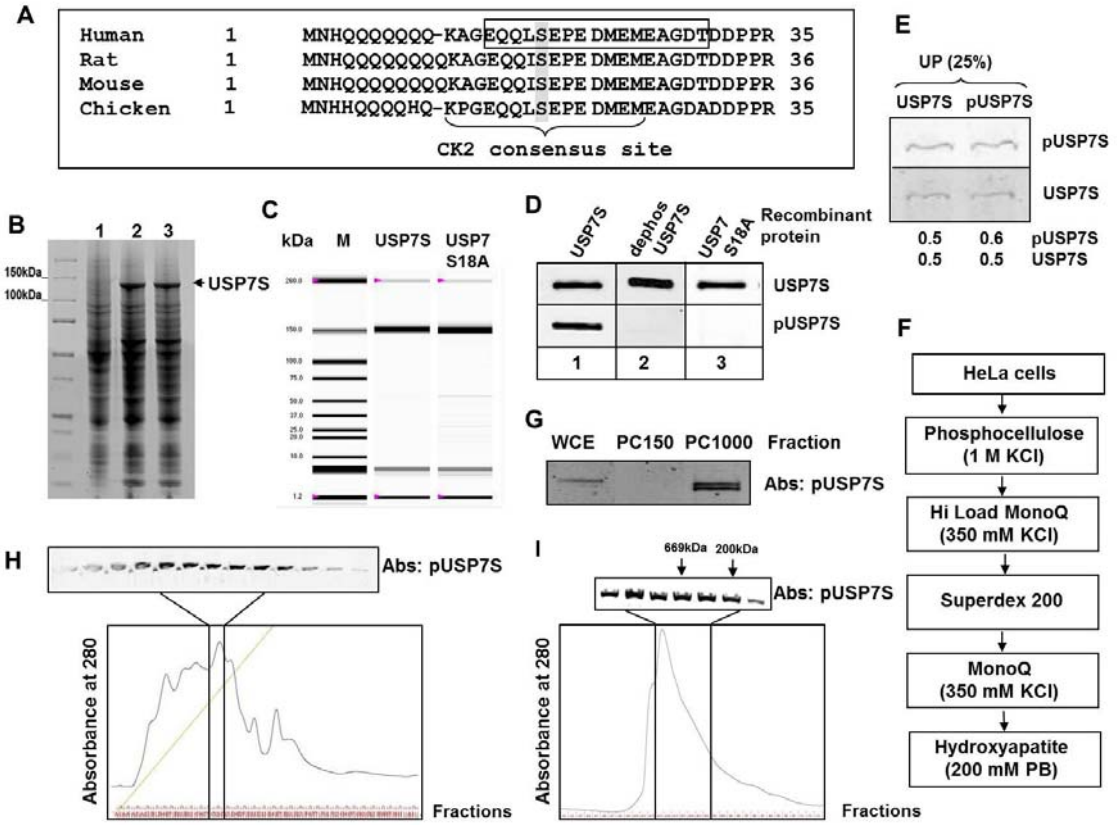


**Figure S1. Expression and Purification of USP7S and USP7S18A from *Sf9* Cells, Specificity of Custom Antibodies which Recognise USP7S Phosphorylated at S18 Residue and Purification of Major Kinase Activity for USP7S from HeLa Cells, Related to Figure 1**

**(A)** N-terminal sequence of USP7S which is highly conservative in mammals. Ser18 is highlighted; CK2 consensus and peptide used to prepare custom antibodies are shown. **(B)** To express recombinant proteins, *Sf9* cells (lane 1) were infected by plaque-purified viral stock and grown in suspension for 72h at 27ºC. Whole cell extracts of USP7S (lane 2) and USP7S18A (lane 3) prepared from *Sf9* cells were loaded onto a HisTrap HP column (GE Healthcare), His-tagged USP7S and USP7S18A were eluted in 250 mM imidazole-containing buffer. **(C)** Electrophoresis of purified proteins in denaturing conditions was performed using Experion automated electrophoresis system (BioRad Laboratories). **(D)** 0.2 pmol of purified recombinant USP7S, dephosphorylated USP7S or mutant USP7S18A were separated on a 10% Tris-Gly gel, followed by transfer to a PVDF membrane and immunobloting with custom USP7S and pUSP7S antibodies. Dephosphorylated USP7S was prepared *in vitro* by incubating recombinant USP7S (1 pmol) with shrimp alkaline phosphatase (1 pmol) at 37ºC for 1h with shaking. Then reaction mixture was loaded onto a 1 mL His-Trap column (GE Healthcare) and His-tagged dephosphorylated USP7S was eluted in buffer containing 250 mM imidazole. **(E)** HeLa whole cell extracts were immunoprecipitated using USP7S or pUSP7S antibodies (**Fig. 1B**) and the unbound proteins (UP, 25 %) were separated by 10 % SDS-PAGE and quantitatively analysed by Western blotting using pUSP7S or USP7S antibodies. **(F)** Purification scheme. **(G)** HeLa whole cell extracts were loaded on Phosphocellulose column in 150 mM salt-containing buffer. The

flow-through (PC150) was collected and proteins bound to the column were eluted with 1 M salt-containing buffer (PC1000), 10 µg of each PC150 and PC1000 fraction were used in an *in vitro* kinase activity assay. **(H)** USP7S kinase activity-containing PC1000 fraction was then subjected to fractionation on 5 mL HiTrapQ HP column. Proteins were eluted using a linear gradient of 50-1000 mM KCl and *in vitro* kinase activity was performed using the obtained fractions. (**I**) Active fractions were pooled and fractionated on a Superdex 200 column prior to analysis using an *in vitro* kinase activity assay.

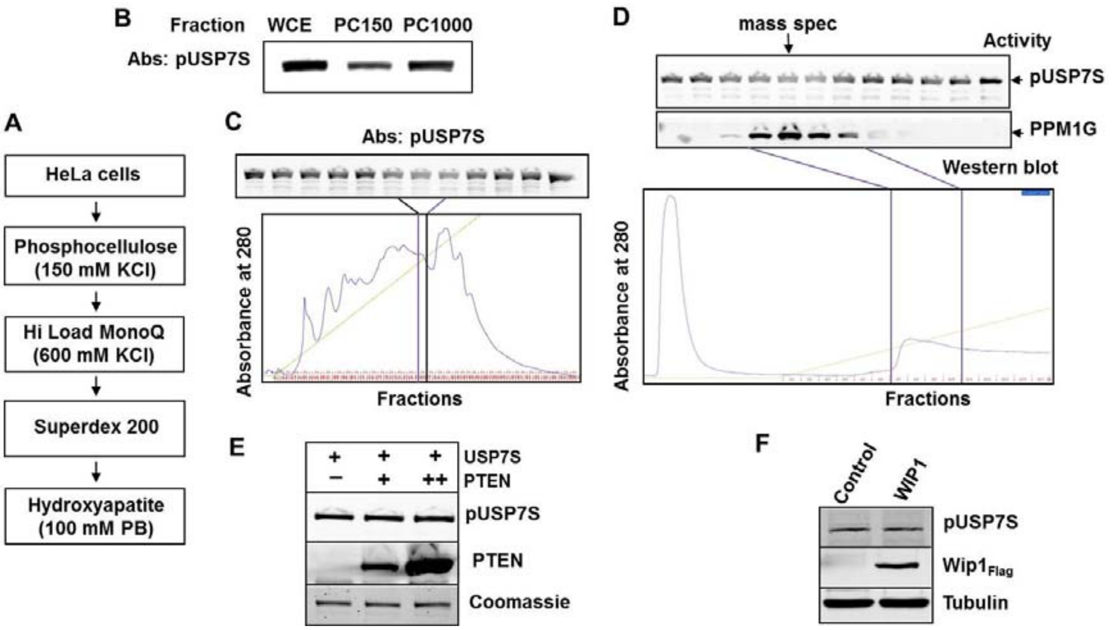


**Figure S2. Purification of Major Phosphatase Activity for USP7S from HeLa Cells and Dephosphorylation of USP7S by PTEN and WIP1, Related to Figure 4**

**(A)** Purification scheme. **(B)** HeLa whole cell extracts were loaded onto a Phosphocellulose column in 150 mM salt-containing buffer. The flow-through (PC150) was collected and 1 M salt-containing buffer was used to elute the proteins bound to the column (PC1000), 10 µg of each PC150 and PC1000 fraction were used in a phosphatase activity assay. (**C**) USP7S phosphatase activity containing PC150 fractions were subjected to fractionation on a 5 mL HiTrapQ HP column. Proteins were eluted using a linear gradient of 50-1000 mM KCl and fractions were used in an *in vitro* activity assay. **(D)** Active fractions were then pooled and fractionated on a Superdex 200 column (shown in **Fig. 4A**) followed by an additional fractionation on a 1 mL hydroxyapatite column and analysed using an *in vitro* phosphatase activity assay. **(E)** *In vitro* dephosphorylation of recombinant pUSP7S (2.8 pmol) by recombinant PTEN (2.8 and 11.2 pmol) was performed at 37ºC for 30 min in buffer containing 10 mM HEPES, 150 mM NaCl, 10 mM DTT, pH 7.2. Substrate equal loading was demonstrated using Coomassie staining. **(F)** U2OS cells were treated with Lipofectamine transfection reagent (10 µl) in the absence (Control) or presence of WIP1 expression vector (2 µg) for a further 24 h. Cells were pelleted by centrifugation, whole cell extracts were prepared and analysed by 10 % SDS-PAGE and Western blotting with the antibodies indicated.

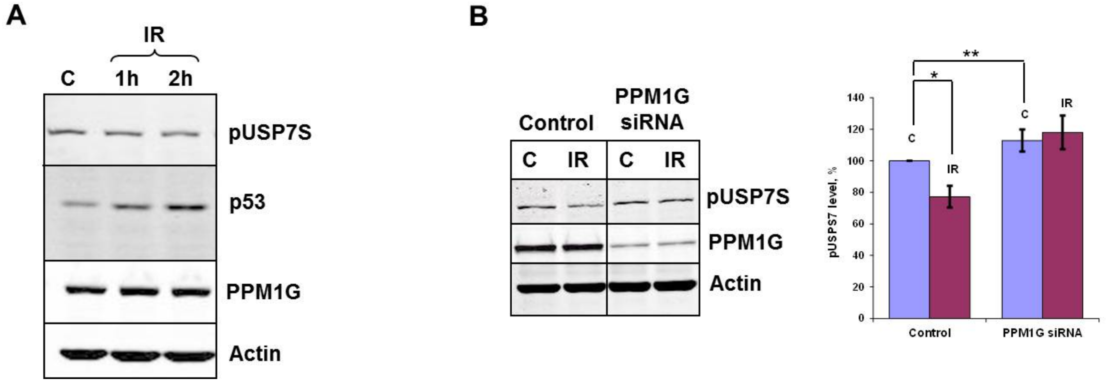


**Figure S3. Knockdown of PPM1G Inhibits IR-Induced Dephosphorylation of USP7S, Related to Figure 5**

**(A)** HCT116 $p53^{+/+}$ cells were grown on 10 $cm^2$ dishes for 24 h to 70-80 % confluency and then either left untreated (C) or exposed to ionizing radiation IR (10 Gy) and harvested at the indicated time points post-treatment. **(B)** HCT116 $p53^{+/+}$ cells (30-50 % confluency) were treated with Lipofectamine RNAiMAX transfection reagent (10 µl) in the absence (Control) or presence of PPM1G siRNA (200 pmol) for a further 72 h. Cells were harvested either without any additional treatment (C) or 1 h post-treatment with ionizing radiation (IR). **(A, B)** Whole cell extracts were prepared and analysed by 10 % SDS-PAGE and Western blotting with the antibodies indicated. Quantitative results are presented as a Mean ± S.D. of 5 biological experiments, where *$p$<0.001, **$p$<0.01 as analysed by Student`s $t$-test.

**Table S1. Identification of the Casein Kinase II (CK2) by Tandem Massspectrometry, Related to Figure 1**

**P19784 CKIIα Casein kinase II subunit alpha**

MPGPAAGSRAR**VYAEVNSLR**SREYWDYEAHVPSWGNQDDYQLVRKLGRGKYSEVFEAIN
ITNNERVVVKILKPVKKKKIKREVK**ILENLR**GGTNIIK**LIDTVKDPVSKTPALVFEYIN**
**NTDFK**QLYQILTDFDIRFYMYELLKALDYCHSKGIMHRDVKPHNVMIDHQQKKLRLIDW
GLAEFYHPAQEYNVRVASRYFKGPELLVDYQMYDYSLDMWSLGCMLASMIFRR**EPFFHG**
**QDNYDQLVR**IAK**VLGTEELYGYLK**KYHIDLDPHFNDILGQHSRKR**WENFIHSENRHLVS**
**PEALDLLDK**LLRYDHQQRLTAK**EAMEHPYFYPVVKEQSQPCADNAVLSSGLTAAR**

Mascot Protein Score: 470

| Peptide Sequence | Mr (exp), [Da] | Mr(calc), [Da] | Delta Mass, [Da] |
|---|---|---|---|
| $_{12}$VYAEVNSLR$_{20}$ | 1049.4 | 1049.5 | -0.1 |
| $_{85}$ILENLR$_{90}$ | 756.5 | 756.4 | 0.1 |
| $_{98}$LIDTVKDPVSK$_{108}$ | 1213.8 | 1213.7 | 0.1 |
| $_{109}$TPALVFEYINNTDFK$_{123}$ | 1771.1 | 1770.9 | 0.2 |
| $_{157}$DVKPHNVMIDHQQK$_{170}$ | 1688.2 | 1687.8 | 0.4 |
| $_{231}$EPFFHGQDNYDQLVR$_{245}$ | 1864.1 | 1863.8 | 0.4 |
| $_{249}$VLGTEELYGYLK$_{261}$ | 1511.1 | 1511.8 | 0.7 |
| $_{282}$WENFIHSENR$_{291}$ | 1330.8 | 1330.6 | 0.2 |
| $_{292}$HLVSPEALDLLDK$_{304}$ | 1448.9 | 1448.8 | 0.1 |
| $_{318}$EAMEHPYFYPVVK$_{330}$ | 1609.0 | 1608.8 | 0.2 |
| $_{331}$EQSQPCADNAVLSSGLTAAR$_{350}$ | 2074.1 | 2073.9 | 0.2 |

Hydroxyapatite fractions displaying major kinase activity against USP7S at Ser18 (**Fig. 1C)** were analysed by tandem mass spectrometry (LC-MS/MS) that revealed the identification of various tryptic fragments matching the protein sequence of the casein kinase II subunit α (SwissProt Accession number P19784).

**Table S2. Identification of the Protein Phosphatase 1G (PPM1G) by Tandem Mass Spectrometry, Related to Figure 4**

**O15355 PPM1G HUMAN Protein phosphatase 1G**

MGAYLSQPNTVK**CSGDGVGAPR**LPLPYGFSAMQGWRVSMEDAHNCIPELDSETAMF
SVYDGHGGEEVALYCAKYLPDIIKDQKAYKEGKLQKALEDAFLAIDAKLTTEEVIK
ELAQIAGRPTEDEDEKEKVADEDDVDNEEAALLHEEATMTIEELLTRYGQNCHKGP
PHSKSGGGTGEEPGSQGLNGEAGPEDSTR**ETPSQENGPTAK**AYTGFSSNSERGTEA
GQVGEPGIPTGEAGPSCSSASDKLPRVAKSKFFEDSEDESDEAEEEEEDSEECSEE
EDGYSSEEAENEEDEDDTEEAEEDDEEEEEEMMVPGMEGKEEPGSDSGTTAVVALI
RGKQLIVANAGDSR**CVVSEAGK**ALDMSYDHKPEDEVELARIKNAGGKVTMDGRVNG
GLNLSRAIGDHFYKRNKNLPPEEQMISALPDIKVLTLTDDHEFMVIACDGIWNVMS
SQEVVDFIQSKISQRDENGELRLLSSIVEELLDQCLAPDTSGDGTGCDNMTCIIIC
FKPRNTAELQPESGKRKLEEVLSTEGAEENGNSDKKKKAKRD

Mascot Protein Score: 140

| Peptide Sequence | Mr (exp), [Da] | Mr(calc), [Da] | Delta Mass, [Da] |
|---|---|---|---|
| $_{13}$CSGDGVGAPR$_{22}$ | 974.433 | 974.424 | 0.009 |
| $_{198}$ETPSQENGPTAK$_{209}$ | 1257.594 | 1257.583 | 0.011 |
| $_{351}$CVVSEAGK$_{358}$ | 848.407 | 848.406 | 0.001 |

Fractions purified from HeLa whole cell extracts and showing phosphatase activity against USP7S at Ser18 residue (**Fig. S4D**) were analysed by tandem mass spectrometry (LC-MS/MS) that revealed the identification of tryptic fragments corresponding to peptides 13-22, 198-209 and 351-358 matching the protein sequence of the protein phosphatase 1G (SwissProt Accession number O15355).

## Supplemental References

Aslanidis, C., and de Jong, P. J. (1990). Ligation-independent cloning of PCR products (LIC-PCR). Nucleic Acids Res *18*, 6069-6074.

Batycka, M., Inglis, N. F., Cook, K., Adam, A., Fraser-Pitt, D., Smith, D. G., Main, L., Lubben, A., and Kessler, B. M. (2006). Ultra-fast tandem mass spectrometry scanning combined with monolithic column liquid chromatography increases throughput in proteomic analysis. Rapid Commun Mass Spectrom *20*, 2074-2080.

Borodovsky, A., Ovaa, H., Kolli, N., Gan-Erdene, T., Wilkinson, K. D., Ploegh, H. L., and Kessler, B. M. (2002). Chemistry-based functional proteomics reveals novel members of the deubiquitinating enzyme family. Chem Biol *9*, 1149-1159.

Taylor, G. K., and Goodlett, D. R. (2005). Rules governing protein identification by mass spectrometry. Rapid Commun Mass Spectrom *19*, 3420.

Wessel, D., and Flugge, U. I. (1984). A method for the quantitative recovery of protein in dilute solution in the presence of detergents and lipids. Anal Biochem *138*, 141-143.
